# Participatory design in the development of the wheelchair convoy system

**DOI:** 10.1186/1743-0003-5-1

**Published:** 2008-01-02

**Authors:** Vinod Sharma, Richard C Simpson, Edmund F LoPresti, Casimir Mostowy, Joseph Olson, Jeremy Puhlman, Steve Hayashi, Rory A Cooper, Ed Konarski, Barry Kerley

**Affiliations:** 1 Department of Bioengineering; University of Pittsburgh; Pittsburgh, PA, USA; 2 Department of Rehabilitation Science and Technology; University of Pittsburgh; Pittsburgh, PA, USA; 3 Human Engineering Research Labs; VA Pittsburgh Healthcare System; Pittsburgh, PA, USA; 4 AT Sciences; Pittsburgh, PA, USA; 5 J. Iverson Riddle Development Center; Morganton, NC, USA

## Abstract

**Background:**

In long-term care environments, residents who have severe mobility deficits are typically transported by having another person push the individual in a manual wheelchair. This practice is inefficient and encourages staff to hurry to complete the process, thereby setting the stage for unsafe practices. Furthermore, the time involved in assembling multiple individuals with disabilities often deters their participation in group activities.

**Methods:**

The Wheelchair Convoy System (WCS) is being developed to allow a single caregiver to move multiple individuals without removing them from their wheelchairs. The WCS will consist of a processor, and a flexible cord linking each wheelchair to the wheelchair in front of it. A Participatory Design approach – in which several iterations of design, fabrication and evaluation are used to elicit feedback from users – was used.

**Results:**

An iterative cycle of development and evaluation was followed through five prototypes of the device. The third and fourth prototypes were evaluated in unmanned field trials at J. Iverson Riddle Development Center. The prototypes were used to form a convoy of three wheelchairs that successfully completed a series of navigation tasks.

**Conclusion:**

A Participatory Design approach to the project allowed the design of the WCS to quickly evolve towards a viable solution. The design that emerged by the end of the fifth development cycle bore little resemblance to the initial design, but successfully met the project's design criteria. Additional development and testing is planned to further refine the system.

## Background

### Problem statement

The number of citizens requiring long-term care will more than double by the middle of this century to 27 million people [1]. Most of this increase will be due to aging baby boomers but also includes a significant increase in adults with various types of disabilities. One of the most important, yet labor intensive, services of personal care is individual mobility. An unpublished survey of three intermediate care facilities (ICFs) for people with developmental disabilities in the State of North Carolina conducted by the J. Iverson Riddle Development Center (JIRDC) found that, of 1410 residents, 655 (46%) had significant mobility deficits that require the use of wheelchairs for travel but only 2% of these individuals were able to use a powered wheelchair independently.

In ICFs, nursing homes, and other long-term care environments, residents who have severe mobility deficits move about by being pushed in a manual wheelchair by a caregiver. Moving a group of residents between locations is labor intensive, and requires at least three caregivers: one to stay with residents at the starting location, a second to stay with residents at the goal location, and a third to move one person at a time from the start location to the goal location.

This practice is inefficient and encourages staff to hurry to complete the process, thereby setting the stage for unsafe practices. Such practices could be responsible for injuries to the residents or the staff (for example, back injuries due to rushed movements and poor ergonomics). Furthermore, the time involved in assembling multiple residents with disabilities often deters their participation in group activities.

Removing the need for the one-to-one method for meeting mobility needs would free staff to meet other personal needs. To estimate savings in staff time, the number of trips made by 6 residents (in varying group sizes) from one of the homes at JIRDC was counted, along with the number of staff required to make each trip. Trips were analyzed from Monday through Friday for a typical week of work and other activities. Weekend trips were not analyzed as they are unpredictable due to the varying activities of the residents. A total of 136 trips were found necessary for the 6 residents to attend their typical weekly vocational and recreational activities. Each person required one staff person to assist in their movement, meaning that 136 staff trips were required to accomplish this travel schedule. If the residents could travel in a group mode when appropriate, such that only one staff person would be needed to accomplish the travel, only 76 staff trips (a 44% reduction) would have been necessary to accomplish the same travel schedule.

One option for group travel is a cart, van, or bus that transports non-ambulatory groups of people around a facility. However, this solution has several drawbacks. For instance, each person in a wheelchair must either use a safety tie-down system or be transferred from their wheelchair to a secure seat. The time required to load and off-load such a vehicle can be significant when working with multiple wheelchair users. Infrastructure must also exist to support access into and near the home pickup points and sufficient tram staff to operate, load, unload and maintain such a system.

An alternative is a system that allows a single staff member to lead a "convoy" of self-propelled wheelchairs. The Wheelchair Convoy System (WCS) will consist of a processor and a physical or virtual "linkage" between the chairs. Initial development was conducted using a power wheelchair and a digital camera, but the most recent prototype is constructed on a JWI power-assist manual wheelchair and uses a mechanical linkage.

### Related research

Several researchers have used technologies originally developed for mobile robots to create *Smart Wheelchairs *[2]. These devices typically consist of either a traditional powered wheelchair to which a computer and a collection of sensors have been added or a mobile robot base to which a seat has been attached. Two North American companies, Applied AI [3] and ActivMedia [4], sell smart wheelchair prototypes based on modified power wheelchair bases, but neither system is intended for use outside of a research lab. Applied AI's TAO-7 makes use of 13 IR sensors, 8 ultrasonic sensors and 1 CCD camera to perform landmark-based navigation. ActivMedia's RoboChariot makes use of shaft encoders and a laser range finder to create a map of its environment, which can then be used for localization and navigation.

The CALL Center of the University of Edinburgh, Scotland, has developed a smart powered wheelchair with bump sensors, sonar sensors, and the ability to follow tape tracks on the floor for use within a wheeled-mobility training program [5]. This chair is sold in the United Kingdom (UK) and Europe by Smile Rehab, Ltd. (Berkshire, UK) as the "Smart Wheelchair." Another relevant product is the "Smart Box," which is also sold by Smile Rehab in the UK and Europe. The Smart Box is compatible with powered wheelchairs using either Penny and Giles or Dynamics control electronics and includes bump sensors (but not sonar sensors) and the ability to follow tape tracks on the floor. Both the Smile Rehab Smart Wheelchair and Smart Box differ from the WCS in that they are intended as mobility training devices rather than mobility aids, both require constant active input from the wheelchair user in order to move, and neither product supports group travel.

Smart wheelchairs have been developed that follow moving targets using a variety of sensing modalities, and could conceivably be used to form wheelchair convoys, though none has been used for this purpose. The INRO smart wheelchair used sonar sensors to track targets in order to form convoys [6]. The Human Tracking and Following System [7] uses a custom-built, highly directional, steerable Wi-Fi antenna to track and follow a person carrying a Wi-Fi enabled pocketPC. The Intelligent Wheelchair System from Osaka University [8] uses two cameras, one facing toward the user and the second facing forward. The user provides input to the system with head gestures, interpreted by the inward-facing camera. The outward-facing camera tracks targets and allows the user to control the wheelchair with gestures when out of the wheelchair. When the user looks straight ahead for a short time, the outward-facing camera identifies the target and moves toward it. Several other smart wheelchairs have also demonstrated target tracking either with [9-13] or without [14,15] machine vision.

## Methods

### Participatory design

**Figure 1:**
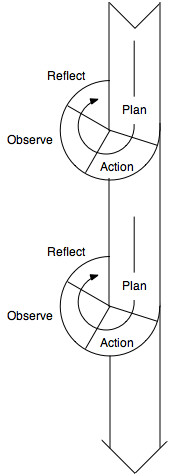
The iterative cycles of action research and participatory design (from [24]).

The development process was guided by the framework of Participatory Design. Based on Action Research [16], the goal of Participatory Design is to produce both new technologies and organizational patterns in close cooperation with end users [17]. A distinguishing feature of Participatory Design is the use of multiple cycles of implementation, adaptation and evaluation driven by stakeholders (as shown in Figure 1).

Participatory Design relies on participants' ability to envision how devices *will look *or *will behave*, often through mock-ups and simulations [17]. Typical methods used to elicit and evaluate ideas include examination of similar products, metaphors, analogies, scenarios, role playing, sketches, models and prototypes [17-20]. One observation from previous Participatory Design efforts is that models and prototypes are more effective than drawings, because they provide physical artifacts with which participants can interact [18]. During the course of development of the WCS, four separate prototypes were constructed and evaluated, each of which is described below. The first two prototypes were based on a powered wheelchair and a laptop to facilitate rapid development. The final two prototypes used a manual wheelchair frame with powered wheelchair hubs.

### Vision-based tracking implemented on a power wheelchair

**Figure 2:**
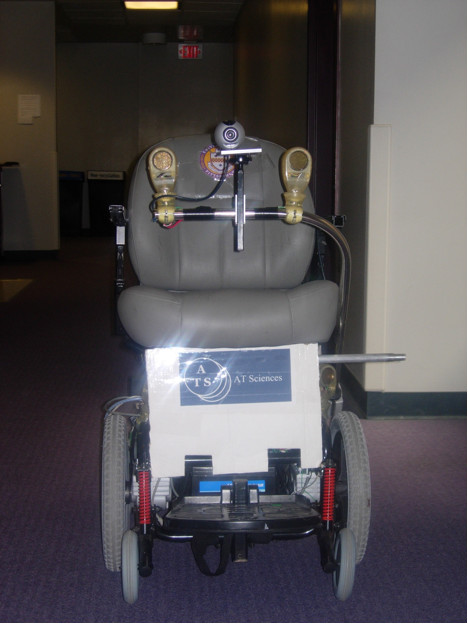
Semi-Autonomous Wheeled Mobility System (Prototype 1).

The initial prototype of the WCS (see Figure 2) was based on a midwheel drive power wheelchair. Three Sonaswitch MiniA sonar sensors and a Logitech Quickcam Pro-4000 camera were mounted on the wheelchair lap tray. The control software was written in Microsoft Visual C++ 6.0 and implemented on a Pentium III, 933Mhz, 528MB RAM Toshiba Laptop. Computer vision was implemented using the Intel OpenCV libraries [21]. Two PCMCIA data acquisition cards were used to interface with the sonar sensors and the wheelchair's motor controller.

**Figure 3:**
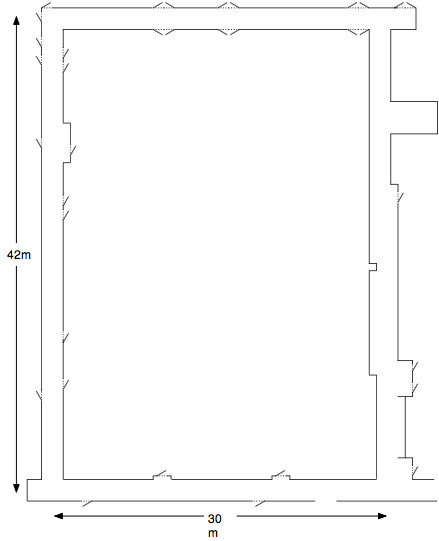
Course navigated by WCS while tracking a moving person.

The system was evaluated in unmanned tests in an office building on the University of Pittsburgh campus. The wheelchair tracked a moving target (one of the investigators wearing a green shirt) around the course shown in Figure 3. The course spans a total distance of 142 meters (155 yards), and includes numerous alcoves and outcroppings. At its narrowest point, the course is 1.4 meters (4.5 feet) wide. No additional lighting beyond the existing ambient light was provided, and one section of the course was particularly dark due to a burned out light fixture. The course was empty of moving objects, other than the investigator. The system successfully navigated the entire course at a constant speed without colliding with an obstacle.

The limiting factors in this prototype of the WCS were the limited field of view of the camera, lack of degrees of freedom in the camera mounting, and the low quality of the image. The field of view and lack of a pan-tilt mechanism combined to place a lower bound on the turning radius the WCS could achieve when tracking a target. If a target moved too far to the side (corresponding to a very tight turn), the target could leave the camera image before the WCS had time to turn. Addressing this would have required replacing the existing camera with a camera that has a wider field-of-view or mounting the camera on a pan-tilt unit.

### Tracking based on a rigid mechanical linkage implemented on a power wheelchair

Based on the shortcomings of the first prototype, an alternative design approach was pursued. The goal was to develop a system that was more reliable than the first prototype and capable of making tight turns. The approach that was decided upon was a physical link between the wheelchairs.

**Figure 4:**
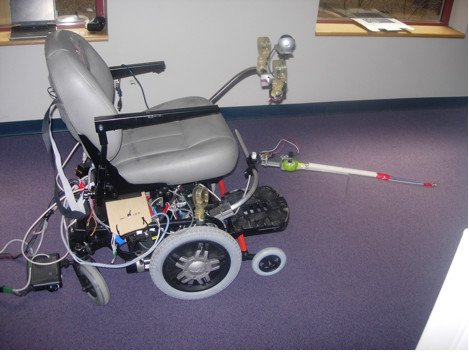
Semi-Autonomous Wheeled Mobility System (Prototype 2).

The second prototype WCS, shown in Figure 4, was implemented using the same power wheelchair and laptop as the first prototype. The second prototype used a tape measure to provide a (semi-)rigid link between the leading and following wheelchair. A rotational encoder was integrated with the tape measure to determine distance between the wheelchairs and a second rotational encoder was used to determine the angle between the wheelchairs.

The system was evaluated in unmanned tests using the same navigation task as the first prototype (see Figure 3). The course was empty of moving objects, other than the investigator. The system successfully navigated the entire course at a constant speed without colliding with an obstacle. The wheelchair did not come to a halt during the trial. The second prototype validated the idea of a mechanical linkage between the wheelchairs. The system was impervious to changes in lighting conditions, and the lead wheelchair never left the trailing wheelchair's "field of view."

### Tracking based on a rigid mechanical linkage implemented on a manual wheelchair

Based on the success of the second prototype, the next step was to implement the system on a manual wheelchair using a more refined mechanical linkage. A manual wheelchair was targeted because that is the type of wheelchair most often used by the target user population of the WCS. Initial designs focused on the Yamaha JWII pushrim-activated power-assist wheelchair hubs [22,23], but it was determined that the motors used by these hubs were not sufficiently powerful to independently travel long distances with a passenger. Instead, Yamaha JWI powered hubs were used. The JWI wheels can be mounted on a manual wheelchair frame and driven with a joystick, without the need for any propulsive force from the wheelchair passenger.

**Figure 5:**
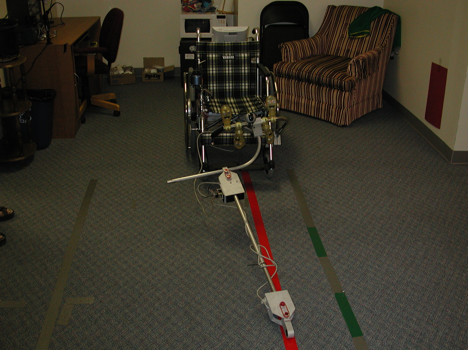
Semi-Autonomous Wheeled Mobility System (Prototype 3).

**Figure 6:**
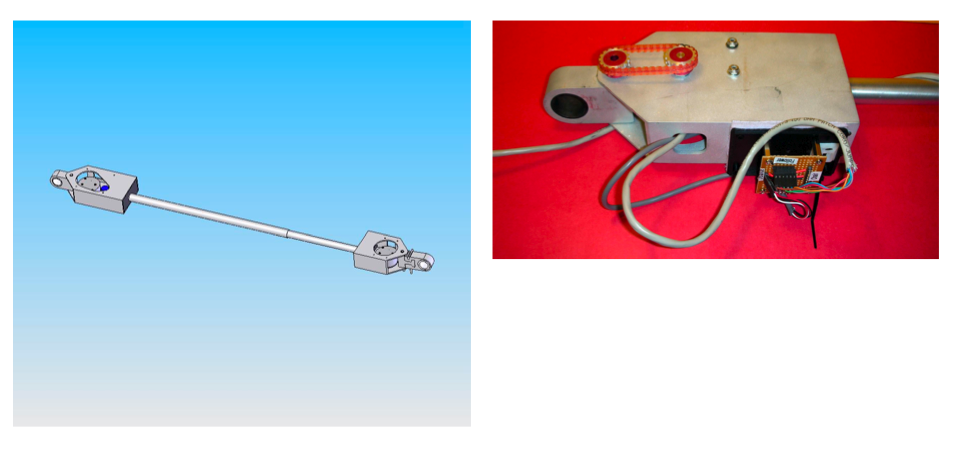
Rigid mechanical linkage used by the third prototype of the WCS. Left panel is a design drawing of the entire linkage. Right panel is a detailed photo of one end of the linkage.

The third prototype of the WCS is shown in Figure 5. The prototype consists of a manual wheelchair frame, a Versalogic computer with built-in A/D and D/A boards and a rigid mechanical linkage for connecting the lead wheelchair to the trailing wheelchair. The computer uses the WindowsXP operating system and the control software was written in Microsoft Visual C++ 6.0. The mechanical linkage (shown in Figure 6) was fabricated from aluminum and consisted of a telescoping bar with clamps on either side. Each clamp contained a rotary encoder, and the telescoping shaft contained a linear encoder, allowing the trailing wheelchair to track the distance and orientation of the lead wheelchair. The connection between the joystick and motor controller for the JWI hubs was interrupted and fed through the Versalogic computer, allowing new motor command signals to be transmitted to the motor controller based on the distance between, and relative orientations of, the two wheelchairs.

The third prototype successfully demonstrated the feasibility of implementing the system on a manual wheelchair, but fell short in other areas. In particular, the rigid mechanical linkage was heavy and the telescoping shaft was unwieldy even when contracted to its shortest length. A final prototype was constructed with the goal of incorporating a completely retractable physical connection between the two wheelchairs.

### Tracking based on a flexible mechanical linkage implemented on a manual wheelchair

**Figure 7:**
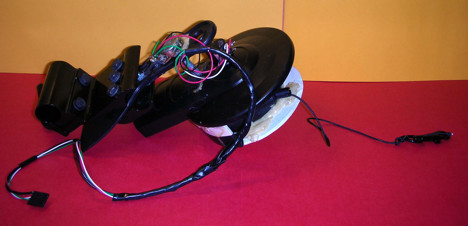
Flexible mechanical linkage used by the fourth prototype of the WCS.

The fourth prototype, shown in Figure 7, was designed based on a retractable string connection (actually, a dog leash) between the two wheelchairs. The same manual wheelchair, JWI hubs and Versalogic computer used by the third prototype were also used. The linkage determined the distance between the wheelchairs based on the number of rotations of the spool holding the string, and determined the angle between the leading wheelchair and trailing wheelchair based on a second encoder connecting the top of the spool to the trailing wheelchair. Unlike the third prototype, there was not an encoder attached to the leading wheelchair and the orientation of the leading wheelchair was therefore not used to control the trailing wheelchair.

As in the third prototype, the connection between the joystick and motor controller for the JWI hubs was interrupted and fed through the Versalogic computer, allowing new motor command signals to be transmitted to the motor controller. The motor command signals were determined based on the distance between the two wheelchairs and the angle of the string connecting them. The distance between the wheelchairs determined the trailing wheelchair's speed, and the angle of the string determined the magnitude of turn.

## Results

**Figure 8:**
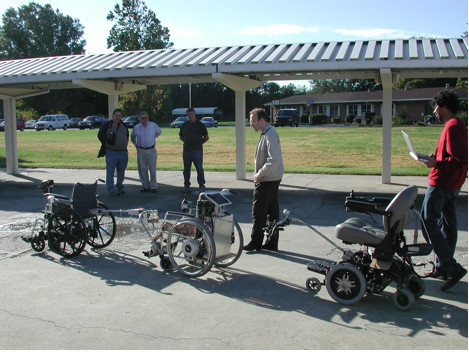
Configuration of wheelchairs for trials conducted at JIRDC.

Unmanned trials of the third and fourth prototypes were conducted on the JIRDC campus. The third prototype was mounted on a manual wheelchair with JWI hubs however, because only one JWI was available for testing, the fourth prototype was mounted on the powered wheelchair used by the first two prototypes. Both chairs were linked together and led by a manual wheelchair to which a powered front tiller (A "Roll-Aid" manufactured by Stand-Aid, Inc. of Iowa) was attached, with the JWI (and third prototype of the WCS) in the middle and the powered wheelchair (and fourth prototype of the WCS) trailing. A picture of the experimental set-up is shown in Figure 8.

### Path navigation

The convoy of all three chairs was tested on a paved path at JIRDC. The path was 164.6 m long, 243.8 cm at its widest point and 172.7 cm at narrowest point. Two laps around the path were completed in 11 minutes and 6 seconds (5:39 for the first lap and 5:27 for the second lap). At no point did any of the wheelchairs stop or leave the path.

### Slalom

The convoy of all three chairs was also tested on a slalom course that was 58.5 m long and 3.7 m wide [Additional file 1]. Each pole on the course was 20.3 cm by 20.3 cm, and 2.2 m separated the front of one pole from the back of the next pole. The convoy of three wheelchairs traversed 11 poles in one direction, turned around, traversed 16 poles in the other direction, turned around again, and traversed an additional 8 poles before the first collision occurred (a total of 35 poles and two complete turns) in 6 minutes and 48 seconds.

## Discussion

The WCS was developed in an iterative fashion, with multiple design and evaluation cycles. Lessons learned during each cycle informed the design goals and evaluation criteria in subsequent cycles. The design criteria that emerged for the WCS by the end of the fourth development/evaluation cycle were:

### Cost

The system must fit within the operating budgets of ICFs. Cost of the system can be amortized by allowing facilities to use the system with multiple residents.

### Compatibility

The system must be compatible with a variety of manual wheelchair models and configurations. Passengers are likely to have a wide variety of seating and positioning needs, which must also be accommodated.

### Simplicity

The system must be easy for caregivers to operate with minimal training. There is significant turnover within ICFs, and staff is often poorly educated.

### Unobtrusiveness

The system must not interfere with normal operation of the manual wheelchair when not in use.

Following the evaluation activities at JIRDC, meetings were held with potential users at two facilities in the Pittsburgh area: Southwestern Veterans Center (SVC) and the Children's Institute (CI). SVC is a residential long-term care facility serving older and disabled veterans in southwestern Pennsylvania. The CI has a day school, outpatient rehabilitation facilities, and a hospital.

Two additional design issues were identified that were not relevant to JIRDC. JIRDC is a large multi-acre campus of single-story buildings, whereas SVC and the CI are both single buildings with multiple floors. The implication for the WCS is that, whereas JIRDC values a solution that can support convoys of multiple wheelchairs traveling long distances, both SVC and the CI need a solution that allows shorter convoys to be quickly assembled and disassembled to allow the use of elevators.

Another issue that was not relevant at JIRDC or SVC, but arose at the CI, was the need to remove the WCS hardware at the end of the day. Residents at JIRDC and SVC rarely leave the facility, so there is no need to remove the WCS hardware. Students at the CI, however, leave each day, and need to be able to use their wheelchairs at home. A solution that works in environments like the CI, therefore, must be easy to attach and detach.

During early iterations of the design/evaluation cycle, a powered wheelchair and laptop computer were used for rapid development and evaluation. The original intent was to eventually transition to a manual wheelchair frame and an embedded processor, and cease development of the WCS for powered wheelchairs. In many cases, particularly for individuals who are not able to operate a manual or powered wheelchair independently, a manual wheelchair frame is desired because it is smaller, lighter and more maneuverable. However, even individuals who are able to operate a powered wheelchair independently might occasionally benefit from the ability to join a convoy of wheelchairs. For example, the WCS may be useful in evacuating an ICF in an orderly manner. Therefore, development of the WCS will continue for both manual wheelchairs (with powered hubs) and traditional powered wheelchairs.

**Figure 9:**
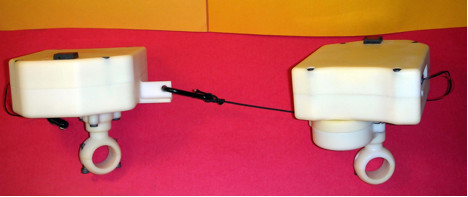
Flexible mechanical linkage developed for fifth prototype of the WCS.

Following testing at JIRDC, a fifth prototype (shown in Figure 9) was developed. Unlike the fourth prototype, the fifth prototype has components on both the leading and trailing wheelchair, so that the trailing wheelchair can use both its orientation and the leading wheelchair's orientation in its navigation calculations. Communication between the two components is wireless, which allows the connection between the two wheelchairs to be an inexpensive string.

**Figure 10:**
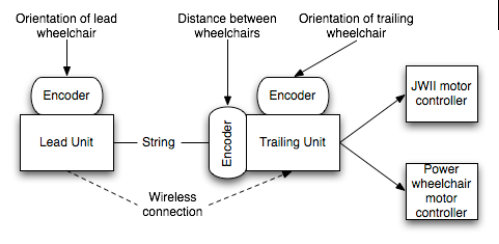
System diagram for fifth prototype of WCS.

A diagram of the current system is shown in Figure 10. The convoy will initially be led by a wheelchair user or by a caregiver holding the first component. Feedback from stakeholders will be used to explore alternatives, including (1) designing a special hand-held component or (2) using computer vision on the first wheelchair in the convoy to track the caregiver.

## Conclusion

Development of the WCS demonstrates the benefits of Participatory Design in the context of developing rehabilitation technology. The original concept of the WCS, based on computer vision and a wireless PDA-based user interface, was dramatically different than the system that was ultimately developed. Frequent evaluations, and interaction with target users at JIRDC, provided valuable guidance for each design cycle.

The WCS is expected to be useful in a variety of settings for both manual and powered wheelchairs. Long-term care facilities with large populations of residents who are not independently mobile are one application of the WCS. Another application of the WCS is within schools serving large numbers of children with disabilities, where the WCS might only be used at the beginning and end of the school day to facilitate bus loading and unloading. Similarly, the WCS might also be useful as a tool for rapid and orderly emergency evacuation of facilities with large numbers of people who use wheelchairs.

## Competing interests

AT Sciences plans to apply for a patent for the Wheelchair Convoy System. AT Sciences will be submitting a Phase I SBIR proposal to the National Institutes of Health based, in part, on the results contained in this manuscript. Dr. Simpson is not employed by, nor does he hold any stocks or shares in, AT Sciences.

## Authors' contributions

RS, EL and RC conceived of the project and participated in its design and coordination. RS drafted the manuscript. VS conceived of the mechanical linkage approach. VS, JO, JP, CM and SH implemented the hardware and software for the system. All authors read and approved the final manuscript. BK and EK provided design feedback throughout the project.

## Supplementary Material

Additional file 1 Three chairs slalom. Video of three wheelchair convoy performing slalom test. Click here for file
